# The Practice Market.—VII

**Published:** 1911-07-15

**Authors:** 


### SPECIAL ARTICLE.

### THE PRACTICE MARKET.—VII.

### THE RELATIONS BETWEEN THE PURCHASER AND THE AGENT.

We now come to the point where the intending purchaser of a practice or a partnership reaches the office of a medical agent. Here it should be stated at once that the agent's first consideration is the vendor, on whose behalf he has undertaken to try and find a suitable purchaser, and from whom he will obtain his commission if he is successful in so doing. The agent is the middleman, and it is in his interest to please both parties; but he naturally wishes to obtain for the seller the best possible price, since he is "the bird in hand." No respectable agent will knowingly touch a bogus practice or intentionally endorse an opening which has been misrepresented; his principle merely is *caveat emptor*, and in this he is supported by the law. Such being the case, the buyer must exercise the greatest caution and suspicion with regard to anything offered him by an agency—he should take nothing on trust; the Practice Market is full of fraudulent goods. An authority on the subject has stated from his certain knowledge—and his experience is enormous—that 75 per cent. of practices negotiated by agents turn out unsatisfactorily to the buyer. And in the same way a practitioner with a really first-rate practice to dispose of, or who wants a first-rate partner, is generally inclined to shun the agencies, at least until he has failed in other directions to find the right man. In justice to the agent, we should add that this is often due to a wish on the vendor's part to avoid the heavy commission *pro rata*; but it reduces the proportion of sound openings available through agencies.

Thus we see that the agent does not deliberately lend himself to misrepresentations; but he makes the best of the information supplied by the vendor in reply to the scheduled questions, without guaranteeing any of his statements or probing below their surface. He has no inducement to direct dishonesty, but rather the reverse; for a bad name is very bad for an agency, and a dissatsified buyer is lost as a client in the future. Nevertheless, the agent is all on the side of the seller, and the range of information which he will give to a prospective buyer about a practice is distinctly limited, and is draped with conventional euphemisms which ring a little hollow in a practised ear. The very best agents are, however, inclined to be less reticent to a *bonâ-fide* inquirer who has been well recommended to them, and in such cases their qualified advice may be of great use to the purchaser.

After the intending buyer has stated his wishes to the agent and filled in the necessary form, he begins to receive from time to time preliminary typewritten particulars of such openings as may be thought likely to interest him. These follow well-accepted lines, and are general in their terms, and non-committal as to the exact locality of the practice. They are chiefly instructive in the points which they omit—these hiatuses soon show up the weak spots in the vendor's case at a glance, and prevent useless inquiries. If, on the other hand, the prospective purchaser is at all favourably impressed with the sketchy information sent to him by an agency in the first instance, he should immediately write for fuller particulars, or whenever possible he should visit the office. Much more can be learnt from one short personal interview with the agent than from a week's correspondence. Almost all agencies make certain stipulations before disclosing the name and address of the vendor, in order to safeguard the latter from injury to his practice, and to his prospects of a satisfactory sale, through his intentions becoming known locally before it is necessary or expedient. The purchaser must, of course, respect these conditions, for without them no agent could retain the confidence of his principal.

The agent does not often hesitate to supply the name and address of the vendor under such conditions, but he may not be so ready to discuss with a possible purchaser any further considerations with regard to the value or suitability of the opening. This reticence on the part of the agent cuts both ways. It is not good business for him to cheapen his client's wares or to suggest difficulties in the way of a bargain. But the best type of applicant on an agent's books is fastidious and wary, and is disinclined to go any further into an opening which is made too much of a secret; he is also unlikely to publish information privately given him by the agent. As a general rule, it may be taken that the better the opening the more expansive will the agent be to a *bonâ-fide* inquirer. If the practice is really sound, undue reticence will injure the vendor's prospects of a good transfer, and will proportionately reduce the agent's commission.

Respectable agencies are always ready to supply a tabulated list of their charges in all ordinary circumstances, as well as full particulars of their methods of business and the exact conditions under which they act for their clients. The prospective buyer pays nothing to the agent for entering his name on the list of applicants, or for the particulars sent to him, or for introductions to vendors, and if a sale results he pays no commission and is not responsible for more than a trifling fee and a share of the cost of an agreement. He cannot therefore expect quite the same zealous care for his interests as the vendor has a right to demand. Nevertheless, good agents do not waste their own or their clients' time with the introduction to each other of medical men whose requirements are obviously irreconcilable. Their object is to bring together at the earliest moment the buyer and seller who are most likely to come to favourable terms and to remain satisfied with their bargain. Our reiterated warnings to those who have dealings with medical agencies must not be taken to imply that we disapprove of the existence of such businesses or condemn their methods. Our criticisms and suggestions do not arise from any hostility to the agents, but from a desire to help practitioners in a vital matter for which their professional upbringing in no way prepares them.

